# PARP-1 Is Critical for Recruitment of Dendritic Cells to the Lung in a Mouse Model of Asthma but Dispensable for Their Differentiation and Function

**DOI:** 10.1155/2019/1656484

**Published:** 2019-04-24

**Authors:** Laura C. Echeverri Tirado, Mohamed A. Ghonim, Jeffrey Wang, Amir A. Al-Khami, Dorota Wyczechowska, Hanh H. Luu, Hogyoung Kim, Maria Dulfary Sanchez-Pino, José Yélamos, Lina M. Yassin, A. Hamid Boulares

**Affiliations:** ^1^The Stanley Scott Cancer Center/Louisiana Cancer Research Center, School of Medicine, Louisiana State University Health Sciences Center, New Orleans, LA, USA; ^2^Grupo de Ciencias Básicas, Escuela de Graduados, Universidad CES, Medellín, Colombia; ^3^Department of Microbiology and Immunology, Faculty of Pharmacy, Al-Azhar University, Cairo, Egypt; ^4^Faculty of Science, Tanta University, Tanta, Egypt; ^5^Cancer Research Program, Hospital del Mar Medical Research Institute, Barcelona, Spain; ^6^Facultad de Medicina, Grupo de Investigaciones Biomédicas Uniremington, Corporación Universitaria Remington, Medellín, Colombia

## Abstract

Dendritic cells (DCs) are critical in asthma and many other immune diseases. We previously demonstrated a role for PARP-1 in asthma. Evidence on PARP-1 playing a role in Th2-associated DC function is not clear. In this study, we examined whether PARP-1 is critical for DC differentiation and function using bone marrow progenitors and their migration to the lung in an ovalbumin-based mouse model of asthma. Results show that changes in PARP-1 levels during GM-CSF-induced DC differentiation from bone marrow progenitors were cyclic and appear to be part of an array of changes that included STAT3/STAT5/STAT6/GRAIL/RAD51. Interestingly, PARP-1 gene deletion affected primarily STAT6 and *γ*H2AX. PARP-1 inhibition significantly reduced the migration of DCs to the lungs of ovalbumin-challenged mice, which was associated with a concomitant reduction in lung levels of the adhesion molecule VCAM-1. The requirement of PARP-1 for VCAM-1 expression was confirmed using endothelial and lung smooth muscle cells. PARP-1 expression and activity were also required for VCAM-1 in differentiated DCs. An assessment of CD11b^+^/CD11c^+^/MHCII^high^ DCs in spleens and lymph nodes of OVA-sensitized mice revealed that PARP-1 inhibition genetically or by olaparib exerted little to no effect on DC differentiation, percentage of CD80^+^/CD86^+^/CD40^+^-expressing cells, or their capacity to promote proliferation of ovalbumin-primed (OTII) CD4^+^ T cells. These findings were corroborated using GM-CSF-induced differentiation of DCs from the bone marrow. Surprisingly, the PARP-1^−/−^ DCs exhibited a higher intrinsic capacity to induce OTII CD4^+^ T cell proliferation in the absence of ovalbumin. Overall, our results show that PARP-1 plays little to no role in DC differentiation and function and that the protective effect of PARP-1 inhibition against asthma is associated with a prevention of DC migration to the lung through a reduction in VCAM-1 expression. Given the current use of PARP inhibitors (e.g., olaparib) in the clinic, the present results may be of interest for the relevant therapies.

## 1. Introduction

Asthma is a serious health issue worldwide as it affects more than 300 million adults and children. A common treatment for asthma is a combination of corticosteroids with a *β*_2_-agonist; however, many patients are refractory to these and other established treatments [1]. Furthermore, although corticosteroids are very effective at blocking asthma-associated Th2 inflammation, the long-term use of these drugs is often associated with many undesired complications that include insulin resistance, type 2 diabetes, osteoporosis, hypertension, and dyslipidemia. One of the primary reasons for these side effects is associated with the potent nonselective immunosuppressive properties of the drugs that affect a litany of important physiological processes [2]. In recent years, much effort has been invested in identifying drug candidates that may target asthma symptoms that are difficult to treat with existing strategies but without causing major immunosuppression. Thus, an examination of the molecular mechanisms that control production of Th2 cytokines and inflammatory factors will undoubtedly increase the likelihood of establishing precise strategies to prevent and/or combat the dire symptoms associated with this disease.

Dendritic cells (DCs) play a pivotal role in the pathogenesis of asthma as they drive the disease through their ability to present antigens and induce primary immune responses in naive CD4^+^ T cells as well as in other Th2 settings [2–4]. DCs also play a key role in non-Th2 responses through mechanisms that involve cytotoxic T cells and other relevant cell types [2–4]. Cell killing associated with DC function is critical to antagonize or block the progression of many cancers [5]. Indeed, DCs are increasingly regarded as a very viable target for therapeutic strategies that aim at enhancing the immune system to fight cancer [5]. Several studies reported changes in the levels of several DNA repair enzymes, such as PARP-1, during the process of DC differentiation from bone marrow progenitors [6] or monocytes [7], suggesting a susceptibility of undifferentiated DCs to DNA-damaging agents. Other studies suggested an important role for PARP-1 in the differentiation and maturation of DCs ([8]; also, see review by Rosado et al. [9]).

Our laboratory conducted a series of pioneering studies that revealed the critical role of PARP-1 in asthma pathogenesis [10–14]. We recently demonstrated that PARP is activated in the lung and peripheral blood mononuclear cells (PBMCs) of asthmatics [15]. We showed that a post-ovalbumin (OVA) challenge administration of a noncompetitive PARP inhibitor, termed thieno [2,3-c]isoquinolin-5-one (TIQ-A), is more efficacious than a prophylactic administration of the drug in reducing OVA-specific IgE production, Th2 responses, and airway resistance in an animal model of asthma [12]. Using the same treatment approach, we recently showed that PARP inhibition by next-generation drugs, such as olaparib (Lynparza™), or gene knockout blocks established asthma-like traits in mice chronically exposed to OVA or house dust mite (HDM) [15, 16]. These effects are linked to a marked reduction in Th2 cytokine production without a prominent effect on Th1 cytokines (e.g., IFN-*γ*) or CD4^+^ T cell proliferation [15]. In a recent study, we showed that PARP-1 inhibition-associated reduction in OVA-specific IgE production can be reversed by adoptively transferring WT OVA-primed (OTII) CD4^+^ Th2-skewed cells into naïve PARP-1^−/−^ mice upon exposure to aerosolized OVA with a complete reversal of IL-4 and GM-CSF [16]. These results suggest to us that PARP-1^−/−^ DCs and B cells are inherently capable of responding to allergen exposure. Given these findings, it became imperative to examine, in detail, the fate of PARP-1 during DC differentiation from bone marrow progenitors and determine whether its inhibition, genetically or pharmacologically by olaparib, influences the differentiation or maturation processes and the capacity of these cells to induce T cell proliferation. Findings of the present study are important in clarifying not only the role of PARP-1 in asthma but also whether therapies that target PARP-1 affect DC differentiation and/or function in patients with cancer.

## 2. Materials and Methods

### 2.1. Animals, Treatments, Tissue Processing, and Immunohistochemistry

C57BL/6J wild type (WT) and B6.Cg-Tg(TcraTcrb)425Cbn/J OTII mice were purchased from The Jackson Laboratory (Bar Harbor, ME, US). C57BL/6 PARP-1^−/−^ mice were described elsewhere [11]. All animals were maintained in a specific pathogen-free facility with unlimited access to sterilized chow diet and water. All protocols were approved by the LSUHSC Animal Care and Use Committee. All animals were genotyped by PCR with DNA extracted from ear punch. Some mice were sacrificed to isolate bone marrow progenitors as described below. Other mice were sensitized *i.p.*, with 100 *μ*g of Grade V chicken OVA (Sigma-Aldrich, St. Louis, MO) mixed with 2 mg of aluminum hydroxide in saline at days 0 and 7. Six hours after the last sensitization, spleens and mesenteric and mediastinal lymph nodes were collected, which were then processed for CD11c^+^ cell isolation. Some sensitized mice were challenged with aerosolized OVA for 30 min, which were sacrificed 24 h later. Lungs were processed to generate single-cell suspensions for staining as described below or fixed for histology or immunohistochemistry. Lung sectioning, staining with hematoxylin and eosin (H&E), and immunostaining with antibodies to mouse VCAM-1 (Santa Cruz Biotechnology, sc-8304) were conducted as described [17]. Immunoreactivity was analyzed using the Image-Pro Plus software (version 6.3) (Silver Spring, MD, USA). The measurement parameters included the density mean and area sum as described previously [18].

### 2.2. Isolation of Bone Marrow Progenitors, Differentiation of Derived Dendritic Cells, Flow Cytometry Analysis, Cell Sorting, and the DC Function Assay

Bone marrow was extracted from the femur and tibia of euthanized WT or PARP-1^−/−^ mice using a syringe-based flushing method. Bone marrow cells were cultured at a density of 2 × 10^5^ cells/ml in RPMI-1640 with L-glutamine and supplemented with penicillin (100 U/ml), streptomycin (100 *μ*g/ml), 2-mercaptoethanol (50 *μ*M), 10% of heat-inactivated fetal calf serum, and 20 ng/ml recombinant mouse GM-CSF. At day 3, an equal volume of the culture medium was added. At day 6, 50% of the medium was replaced with fresh complete medium containing GM-CSF. Some WT cells were treated with GM-CSF in the presence of 1 *μ*M olaparib (AZD2281, Lynparza™) (Selleckchem S1060) or vehicle. The drug was added with every media change. On day 8, nonadherent bone marrow-derived DCs (BMDCs) were evaluated for cell viability with Annexin-V Apoptosis Detection Kit-FITC (eBioscience, San Diego, CA, USA).

The gating strategy was conducted essentially as outlined in the detailed study by Helft et al. [19] using 100,000 events, also see supplementary Figure S1. BMDCs were then phenotyped by flow cytometry with the following fluorescently labeled antibodies (all purchased from BD Biosciences, San Diego, CA, USA): CD11c-APC (HL3 clone), CD11b-PE-Cy™7 (M1/70 clone), MHCI-PE (KH95 clone), MHCII-PerCP-Cy™5.5 (M5/114.15.2 clone), CD40-BV421 (3/23 clone), CD80-FITC (16-10A1 clone), and CD86-BV711 (GL1 clone). MHCII^high^ cell population was determined based on the coexpression of CD11b, CD11c, and MHCII markers. CD11c^+^ cells from spleens of OVA-sensitized mice were phenotyped using the above-described panel of antibodies in addition to CD11c-APC (N418 clone). Lungs from OVA-sensitized and OVA-challenged mice were dissociated to obtain a single-cell suspension as described above and were stained in a similar manner with antibodies to mouse CD45, CD11c, and CD11b. Forward scatter and side scatter plots were used to concentrate on the population of interest and remove debris. Next, FSC Tof/FSC peak and SSC Tof/SS peak doublet discrimination gates were used to concentrate only on single cells. The population of interest was determined based on the coexpression of CD11c and MHCII markers [AS] and further coexpression of the abovementioned markers.

For sorting, cells were stained with a set of fluorescently labeled antibodies (CD11cAPC, CD11b-PE-Cy™7, and MHCII-PerCP-Cy™5.5) and CD11c^+^CD11b^+^MHCII^high^ cells and then sorted with a BD FACSAria. These cells were pulsed with OVA 323-339 peptide (InvivoGen, San Diego, CA, USA) (1 *μ*g/ml^−1^) or dH_2_O (control vehicle) overnight and then cocultured with CFSE-stained CD4^+^ T cells isolated from OTII mice for four days. Purity of CD4^+^ T cells was confirmed as described [17]. For ex vivo cultures, mice were sensitized *i.p.* with 100 *μ*g of Grade V chicken OVA (Sigma-Aldrich, St. Louis, MO) mixed with 2 mg of aluminum hydroxide once per week for two weeks as described [17]. Spleens and mesenteric and mediastinal lymph nodes were collected six hours after the last sensitization and processed for single-cell suspension. Positively selected CD11c^+^ cells (Stem Cell Technologies, Vancouver, Canada) were cocultured with OTII CFSE-stained CD4^+^ T cells for four days. Proliferation of T cells was assessed by flow cytometry; gating strategy and representative histograms depicting T cell proliferation are shown in supplementary Figure S2.

### 2.3. Cell Culture, Protein Extraction, Immunoblot Analysis, RT-PCR, and the Poly(ADP-Ribosyl)Ation Assay

Splenocytes were collected after treatments, and pellets were processed for immunoblot analysis [20]. Immortalized cardiac PARP-1^−/−^ endothelial cells were described in detail by Carrillo et al. [21]. Isolation and culture of lung smooth muscle cells were conducted essentially as described [17]. Transduction of cells with the human PARP-1-encoding adenoviral vector is described [22]. Nitrocellulose membranes were probed with antibodies to PARP-1 (Santa Cruz Biotechnology, sc-8007), STAT6 (Santa Cruz Biotechnology, sc-621), p38 MAPK (Cell Signaling Technology, 9212), GRAIL/RNF128 (Novus Biologicals, NBP2-24610), STAT5 (Santa Cruz Biotechnology, sc-835), STAT3 (Cell Signaling Technology, 9132), RAD51 (Santa Cruz Biotechnology, sc-398587), *γ*H2AX (Cell Signaling Technology, 9718), mouse VCAM-1 (Santa Cruz Biotechnology, sc-8304), or actin (Santa Cruz Biotechnology). The signal was detected using chemiluminescence reagents (Thermo Fisher Scientific, Waltham, MA).

For the poly(ADP-ribosyl)ation assay, recombinant PARP-1 (100 ng, Active Motif, Carlsbad, CA) was incubated for 30 minutes at 37°C in a reaction buffer containing 10 *μ*g/ml sheared DNA (Sigma, D7656) and 2 mM NAD+ (Abcam, ab120403) as described [23] in the presence or absence of olaparib. The reactions were terminated by the addition of SDS sample buffer. Proteins were then subjected to immunoblot analysis with antibodies to poly(ADP-ribose) polymer (Trevigen, Gaithersburg, MD, 4335-MC-100).

Total RNA was extracted from cells and was reverse-transcribed as described [18]. The resulting cDNA was subjected to conventional PCR with primer sets (IDT, San Jose, CA, USA) specific to mouse VCAM-1, mouse inducible NO synthase (iNOS), human PARP-1, *β*-actin, or GAPDH (Supplementary Table 1).

### 2.4. Data Analysis

The PRISM software (GraphPad, San Diego, CA, USA) was used to analyze the differences between experimental groups. Results were expressed as mean ± SD and analyzed by one-way analysis of variance (ANOVA) and Tukey's multiple comparison posttest. Experiments were conducted at least 3 times.

## 3. Results and Discussion

### 3.1. Changes in PARP-1 Protein Levels Are Cyclic during GM-CSF-Induced DC Differentiation from Bone Marrow Progenitors, and Activation by OVA Does Not Alter Such Expression in Mature DCs

**Figure 1:**
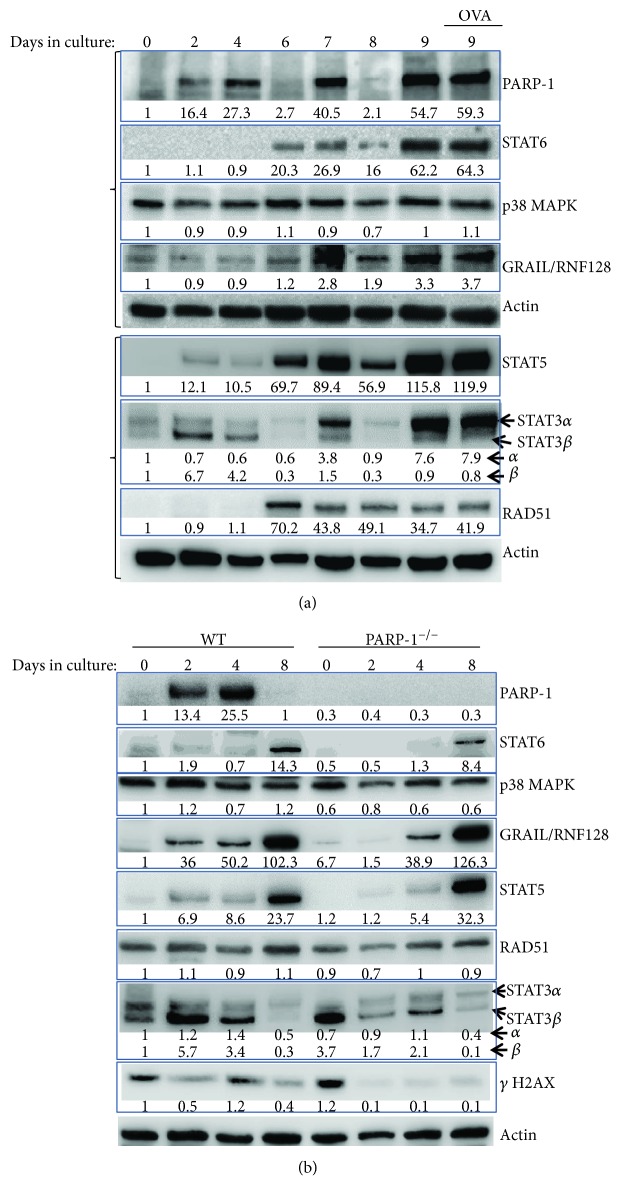
Bone marrow progenitors were isolated from C57BL/6 mice and incubated with 20 ng/ml GM-CSF. (a) Cells were collected at days 0, 2, 4, 6, 7, 8, or 9. A portion of the cells at day 8 were stimulated with OVA for 24 h. Collected cells were processed for protein extraction followed by immunoblot analysis with antibodies to the indicated proteins. Blots were stripped of antibodies prior to probing with the next one. The two braces on the left represent two different gels of the same samples. (b) Bone marrow progenitors from WT or PARP-1^−/−^ mice were isolated and processed as in (a). Protein extracts were subjected to immunoblot analysis with antibodies to the indicated proteins. For (a, b), signals were quantified and are shown as values under the respective blots.

Despite the critical role of dendritic cells in Th2 inflammation and the manifestation of asthma traits, the role of PARP-1 in the differentiation of these cells from bone marrow progenitors and the subsequent antigen presentation remains unsettled. PARP-1 protein was shown to be absent in human monocytes; however, expression of the protein emerged after several days upon treatment with GM-CSF and IL-4 or GM-CSF alone [7]. Given that these findings do not necessarily apply to DCs that originate from the bone marrow, we examined the dynamics of PARP-1 protein expression during the process of GM-CSF-driven differentiation of DCs.  Figure 1(a) shows that bone marrow cells freshly isolated from C57BL/6J WT mice do not express PARP-1, which is consistent with the absence of the protein in monocytes prior to their differentiation to either DCs or macrophages [7]. PARP-1 protein began appearing at day 2 and continued to increase at day 4 of GM-CSF stimulation. Although ultimately (at day 9), PARP-1 levels progressively increased; the process was interrupted by a complete disappearance of the protein at day 6 and day 8. It is noteworthy that GM-CSF was supplemented at day 3 and day 6, which may suggest that the persistence of PARP-1 expression depended on the constant signal from GM-CSF. Interestingly, the appearance of PARP-1 at day 9 occurred without additional supplementation of GM-CSF may negate the latter assessment. Activation of DCs (~85% CD11c^+^) at day 8 with OVA did not induce additional expression of PARP-1. Many proteins were shown to be absent in bone marrow cells but appear during the process of differentiation [7, 8].  Figure 1(a) shows that STAT6, STAT5, STAT3, and RAD51 were all either absent or at very low levels at the day of cell isolation from the bone marrow (day 0) but started appearing at varying rates and stages.

These findings are consistent with published results on bone marrow cells stimulated with GM-CSF and IL-4 [24] or GM-CSF alone [24, 25]. Of note, GRAIL (Gene Related to Anergy in Lymphocytes), a ubiquitin E3 ligase also known as RNF128, which was initially shown to be expressed during the induction of CD4^+^ T cell anergy [26], displayed a pattern of expression similar to the aforementioned proteins. It is rather puzzling to find that expression of GRAIL coincided with that of STAT6. Sahoo et al. [27] showed that GRAIL negatively regulates STAT6 expression and activity as GRAIL gene knockout was accompanied by an increase in STAT6 protein levels with a concomitant promotion of Th2 cytokine production and eosinophilia. One would have predicted that, at least, when GRAIL was absent in the early stages of DC differentiation, STAT6 would have been in its highest levels. Obviously, more experimentation is needed to understand the connection between GRAIL and STAT6 integrity.

An additional expression pattern worth noting is that of STAT3. This transcription factor exists in two isoforms: *α* and *β*. While STAT3*β* appeared early (day 2), STAT3*α* became the predominant isoform at day 9 and remained after activation with OVA. This observation is rather interesting given that STAT3 appears to play a positive role in Flt3L-driven DC differentiation from BM progenitors [28] while it is a negative regulator of splenic DC function with STAT3 conditional knockout mice exhibiting a mild inflammatory phenotype [25]. The two isoforms of STAT3 were reported to display different functions and subcellular dynamics. Upon activation, STAT3*α* appears to be the primary driver of transcription while STAT3*β* exhibits more of a repressor function [29]. Interestingly, while STAT3*β* displays a more persistent nuclear retention, the nuclear localization of STAT3*α* is rather transient [29]. The predominance of STAT3*α* in our experimental model at day 9 and after OVA stimulation is consistent with the notion that the generated DCs are more active. Overall, our results suggest that the changes in PARP-1 protein are part of changes of a litany of proteins that take place during the process of DC differentiation. It is not clear whether these changes are coordinated to achieve some specific status in DC homeostasis or are simply to prepare the cells to function properly upon exposure to a variety of antigens, cytokines, or pathological and environmental insults.

To determine whether PARP-1 influences the expression levels or rates of the assessed proteins, we examined the fate of these proteins during the differentiation process of bone marrow-derived PARP-1^−/−^ DCs.  Figure 1(b) shows that PARP-1 gene deletion exerted little to no effect on most proteins, suggesting that PARP-1 may not be critical for the overall expression of these proteins. The only exception was STAT6, which appears that its levels were reduced in PARP-1^−/−^ DCs at day 8. This result is consistent with our report demonstrating that the integrity of STAT6 may be compromised in PARP-1^−/−^ mice and cells in Th2 conditions [14] and that of Zaffini et al. showing a decrease in STAT6-DNA binding activity in lungs of HDM-challenged mice that were treated with PARP inhibitors compared to mice that did not receive the drugs [30]. It is not clear whether this relationship influenced the differentiation process of bone marrow-derived DCs. Given that PARP-1 is a DNA repair enzyme and the reports that bone marrow progenitors may exhibit a lower capacity in repairing DNA, we assessed whether PARP-1 gene deletion altered the pattern of the phosphorylated form of H2AX (termed *γ*H2AX), a marker of DNA damage response.  Figure 1(b) shows that *γ*H2AX levels were relatively cyclic during the differentiation period of WT progenitors appearing at day 0 and day 4 and disappearing at day 2 and day 8. Interestingly, the levels of the phosphorylated histone in PARP-1^−/−^ DCs appeared at day 0 but disappeared after that at days 4 and 8. These results suggest that the levels of DNA damage were low or absent during the differentiation process of PARP-1^−/−^ DCs. However, the patterns of *γ*H2AX expression are inconsistent with the relatively modest variability of RAD51 levels in WT and PARP-1^−/−^ DCs. Therefore, it is unlikely that the absence of PARP-1 affects the process of DNA break responses during DC proliferation and differentiation. It is important to note that H2AX is phosphorylated primarily by DNA-PK, ATM, or ATR [31]. PARP-1 has been shown to influence the function of these kinases [32, 33], and its absence may affect the phosphorylation status of the histone. Given the redundancy in DNA repair processes, it is unlikely that the changes in H2AX phosphorylation would dramatically affect DC differentiation. However, it is important to acknowledge that our observations do not contradict the studies reporting that DC progenitors may be highly sensitive to DNA-damaging agents [6, 8]. Collectively, these results raise an important question on whether PARP-1 plays a critical part in DC differentiation from bone marrow progenitors.

### 3.2. PARP-1 Inhibition by Gene Deletion or Pharmacologically by Olaparib Exerts Little to No Effect on In Vitro DC Differentiation of Myeloid Progenitors

**Figure 2:**
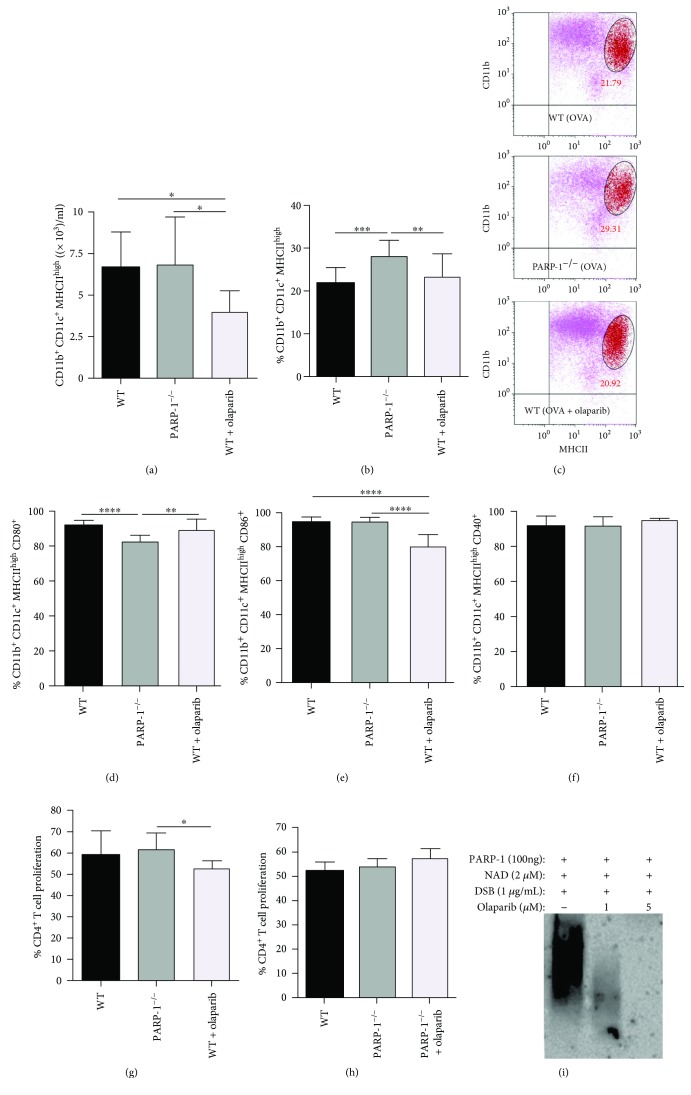
Bone marrow cells isolated from WT or PARP-1^−/−^ mice were cultured in complete medium with 20 ng/ml GM-CSF. WT cells were treated with GM-CSF in the presence of 1 *μ*M olaparib (AZD2281) or vehicle. The drug was added with every media change. On day 8, nonadherent bone marrow-derived DCs were phenotyped by flow cytometry with the fluorescently labeled antibodies CD11c-APC, CD11b-PE-Cy™7, MHCII-PerCP-Cy™5.5, CD40-BV421, CD80-FITC, and CD86-BV711. (a) The number of CD11b^+^/CD11c^+^/MHCII^high^ DCs per ml of culture medium in the different experimental groups. (b) Percent of CD11b^+^/CD11c^+^/MHCII^high^ DCs in the different experimental groups. (c) Representative FACS dot plots of the groups from (b). Percent of CD11b^+^/CD11c^+^/MHCII^high^ DCs expressing CD80 (d), CD86 (e), or CD40 (f). (g–h) Sorted CD11c^+^/CD11b^+^/MHCII^high^ cells from the different experimental groups were pulsed with OVA 323-339 peptide or vehicle overnight, washed, and then cocultured with CFSE-stained CD4^+^ T cells from OTII mice for four days. A portion of CD4^+^ T cells were stimulated with a combination of anti-CD3 anti-CD28 antibodies as a positive control. Proliferation was assessed by flow cytometry. For (a, b) and (d–h), the results are expressed as mean ± SD; ^∗^ *p* ≤ 0.05; ^∗∗^ *p* ≤ 0.01; ^∗∗∗^ *p* ≤ 0.001; ^∗∗∗∗^ *p* ≤ 0.0001. (h) Recombinant PARP-1 was incubated with NAD in the presence or absence of olaparib and activated with double-stranded DNA breaks (DSB) for 30 min. Reactions were stopped by SDS sample buffer and subjected to immunoblot analysis with antibodies to poly(ADP-ribose) (PAR). The smear-like band is typical in poly(ADP-ribosyl)ation reactions showing PARP-1 with different levels of automodification.

To address the above raised hypothesis, we examined whether PARP-1 gene deletion affects the differentiation process of DCs. To this end, myeloid progenitors derived from the bone marrow of WT or PARP-1^−/−^ mice were stimulated with GM-CSF, and the numbers of CD11b^+^/CD11c^+^/MHCII^high^ cells were assessed by flow cytometry after 8 days of culture. For these experiments, we also included cells that were treated with 1 *μ*M of the clinically approved PARP inhibitor olaparib; the drug was replenished every three days. PARP-1 gene knockout exerted no effect on the number of CD11b^+^/CD11c^+^/MHCII^high^ cells compared to the WT counterparts (Figure 2(a)). Interestingly, the percentage of these cells was slightly increased in PARP-1^−/−^ DCs but unaffected in olaparib-treated WT cells (Figures 2(b) and 2(c)). Representative dot plots for the data displayed in Figures 2(d)–2(f) are shown in supplementary Figure S3. The increase in the MHCII^high^ PARP-1^−/−^ cell populations was mirrored with a slight, but statistically significant, decrease in the percentage of MHCII^interm^ (intermediate) PARP-1^−/−^ cells compared to that of WT or olaparib-treated WT cells (Supplemental Figure S4). Although it is difficult to speculate on the reason(s) for such difference, it is plausible that PARP-1 protein, in addition to its activity, plays an additional role in the maturation process of DCs. PARP-1 has been shown to function independently of its poly(ADP-ribosyl)ation activity in several processes [9]. An assessment of the costimulatory markers CD80, CD86, and CD40 in the different experimental groups revealed that the percentage of CD11b^+^/CD11c^+^/MHCII^high^/CD80^+^ cells was slightly reduced in PARP-1^−/−^ but unaffected in olaparib-treated WT DCs compared to the WT counterparts that were not treated with the drug (Figure 2(d)). Interestingly, however, while the percentage of CD11b^+^/CD11c^+^/MHCII^high^/CD86^+^ DCs was unaffected by PARP-1 gene deletion, it was decreased in WT DCs that were treated with olaparib (Figure 2(e)). The frequency of CD11b^+^/CD11c^+^/MHCII^high^ CD40^+^ cells remained the same in all experimental groups (Figure 2(f)). To determine the consequences of these changes, we examined their effects on the capacity of DCs to induce proliferation of CD4^+^ T cells derived from OVA-primed (OTII) mice.  Figure 2(g) shows that PARP-1 gene deletion did not affect the capacity of DCs to induce WT CD4^+^ T cell proliferation despite the slight decrease in the percentage of CD80^+^ cells as indicated in Figure 2(d); however, the reduction of CD86^+^ DCs caused by olaparib treatment (Figure 2(e)) coincided with a decreased capacity of DCs to induce T cell proliferation by OVA-antigen presentation.

The effect of the PARP inhibitor olaparib on DCs expressing CD86 is consistent with that reported by Cavone et al. [34] using GM-CSF-induced mouse DCs differentiated from myeloid progenitors and by Aldinucci et al. [35] using human GM-CSF and IL-4-induced DCs differentiated from monocytes; however, the effects on CD80^+^ DCs and overall CD11c^+^ populations are not. It is noteworthy that the effects on frequency of CD80^+^ DC population observed by the aforementioned studies were attained using very high concentrations of the PARP inhibitors (20-30 *μ*M), such as TIQ-A, and thus, it is conceivable that they are nonspecific and may not be related to the role of PARP-1 in DC function. The slight decrease in CD4^+^ T cell proliferation stimulated by olaparib-treated WT DCs (Figure 2(g)) may not be associated with an effect on PARP-1 as PARP-1^−/−^ DCs were fully functional, and the proliferation of CD4^+^ T cell was not affected when PARP-1^−/−^ DCs were treated with olaparib (Figure 2(h)). Other studies reported substantial reduction in the capacity of differentiated DCs to induce T cell proliferation; nevertheless, these effects may be related to the high concentrations of the PARP inhibitors used and unlikely to be related to an experimental system that focused on Th1 responses [34]. According to Scott et al. [36], PARP inhibition by PJ34 (up to 1 *μ*M) did not affect myelin basic protein- (MBP-) specific T cell proliferation *in vitro*; however, the drug did reduce proliferation of T cells by splenic antigen-presenting cells that were isolated from the same animals. Interestingly, antigen presentation was unaffected in antigen-presenting cells derived from mice that were treated with the PARP inhibitor. Overall, we are confident of our results because we purposely used a combination of genetic and pharmacological approaches to reach our conclusions. The olaparib concentration used in our studies is sufficient to almost completely block DNA break-induced PARP-1 activation *in vitro* (Figure 2(i)). Furthermore, we reported in an earlier study that 0.5 *μ*M TIQ-A is sufficient to almost completely block PARP-1 activation in a cell-free system [37]. The overall conclusion here is that PARP-1 plays little to no role in DC function *in vitro*.

### 3.3. PARP-1 Inhibition by Gene Knockout Reduces DC Migration to Lungs of OVA-Sensitized and OVA-Challenged Mice but Not to Spleens and Lymph Nodes

**Figure 3:**
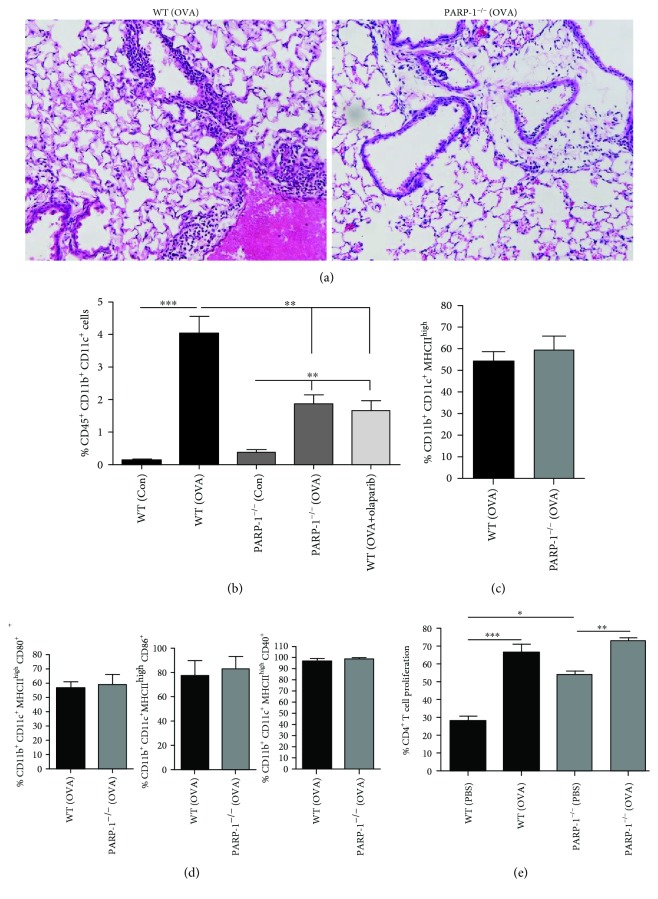
WT or PARP-1^−/−^ mice were subjected to OVA sensitization followed by a single challenge or left unchallenged. A group of mice received olaparib (5 mg/kg) 30 minutes post-OVA challenge. Mice were sacrificed 48 h later. Lungs from the different experimental groups were fixed with formalin or processed to generate single-cell suspensions. (a) Lung sections were stained with hematoxylin and eosin; bar: 50 *μ*m. (b) Cells were stained with a combination of antibodies to CD45, CD11b, and CD11c. CD11b^+^/CD11c^+^ cell population was gated from the live CD45^+^ population. (c) WT or PARP-1^−/−^ mice were sensitized twice with OVA; spleens and lymph nodes were then collected 6 hours after the last sensitization and processed for single-cell suspensions. Cells were then stained with a combination of antibodies to CD11b, CD11c, and MHC. (d) Percentage of CD11b^+^/CD11c^+^/MHCII^high^ DCs that express CD80, CD86, or CD40. (e) Sorted CD11c^+^/CD11b^+^/MHCII^high^ cells from OVA-sensitized WT or PARP-1^−/−^ mice were pulsed with OVA 323-339 peptide or vehicle overnight, washed, and then cocultured with CFSE-stained CD4^+^ T cells from OTII mice for four days. Proliferation was assessed by flow cytometry. For (a, b) and (d–h), the results are expressed as mean ± SD; ^∗^ *p* ≤ 0.05; ^∗∗^ *p* ≤ 0.01; ^∗∗∗^ *p* ≤ 0.001.

Although the above ex vivo results suggest the lack of a key role for PARP-1 in Th2-associated DC differentiation and activation, they may not reflect what actually occurs in a whole-body system, especially in response to allergen exposure. What we know is that PARP-1 inhibition reduces OVA-specific IgE production and that such effect can be mostly reversed by adoptively transferring WT OVA-primed (OTII) CD4^+^ Th2-skewed cells into naïve PARP-1^−/−^ mice upon exposure to aerosolized OVA with a complete reversal of IL-4 and GM-CSF [16]. These results suggest that PARP-1^−/−^ B cells and APCs including DCs are inherently capable of responding to allergen exposure. However, given that PARP-1 inhibition reduces IgE production, we speculated that this effect may be associated with a deficiency in DC migration to the lung rather than in their function. To address this possibility, we assessed DC populations in lung OVA-sensitized and OVA-challenged mice.  Figure 3(a) shows that overall lung inflammation was substantially low in lungs of OVA-sensitized and OVA-challenged PARP-1^−/−^ mice compared to the WT counterparts.  Figure 3(b) shows that OVA sensitization and exposure substantially increased the percentages of lung CD45^+^CD11b^+^CD11c^+^ DCs. The percentages of DCs in OVA-challenged mice are similar to those reported by Mesnil et al. using an HDM-based model of the disease [38]. PARP-1 gene knockout partially reduced (~50%) recruitment of CD45^+^CD11b^+^/CD11c^+^ DCs. Similar reduction in DC recruitment was observed in OVA-sensitized WT mice that received olaparib (5 mg/kg) 30 min after OVA challenge (Figure 3(a), rightmost panel). Contrary to this effect, similar methods of PARP-1 inhibition almost completely blocked migration of other inflammatory cells including eosinophils and lymphocytes as reported in our earlier studies [15, 16]. We next sought to examine whether PARP-1 gene deletion affected the early mobilization and activation capacity of DCs upon OVA sensitization. To this end, WT or PARP-1^−/−^ mice were sensitized twice with OVA as described above; spleens and lymph nodes were then collected six hours after the last sensitization and analyzed for the presence of DC populations by flow cytometry. Interestingly, the percentages of CD11b^+^/CD11c^+^/MHC^high^ DCs in the spleen and lymph nodes of OVA-sensitized PARP-1^−/−^ mice did not differ from that of the WT counterparts (Figure 3(c)). Furthermore, the percentage of CD11b^+^/CD11c^+^/MHC^high^ DCs that express the costimulatory molecules CD80, CD86, or CD40 was equally similar between the two groups (Figure 3(d)). These results are relatively consistent with those attained ex vivo, except for the slight decrease in the percentage of CD80^+^ DCs (Figure 2(d)).

We then examined whether PARP-1 gene deletion affected the capacity of these OVA-primed DCs to induce proliferation of WT OTII CD4^+^ T cells with or without *ex vivo* OVA challenge. Consistent with our *in vitro* results, PARP-1 gene deletion did not affect DC-induced proliferation of T cells when rechallenged with OVA (Figure 3(e)). Surprisingly, however, the intrinsic capacity of PARP-1^−/−^ DCs to induce T cell proliferation was significantly higher, rather than lower, than their WT counterparts.

### 3.4. PARP-1 Inhibition Reduces VCAM-1 Expression in Endothelial and Lung Smooth Muscle Cells

The transendothelial migration of DCs during asthma as well as other inflammatory diseases is largely dependent on the expression of adhesion molecules such as VCAM-1 [3, 39].

**Figure 4:**
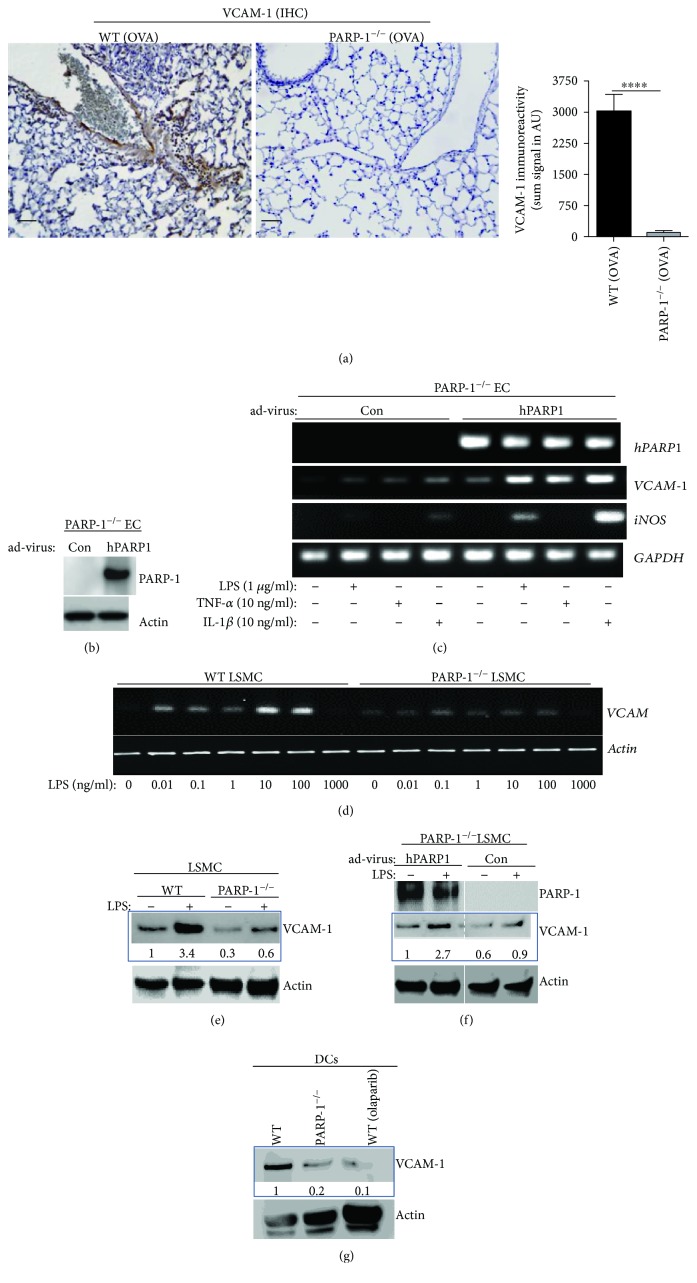
(a) Lung sections from OVA-sensitized and OVA-challenged WT or PARP-1^−/−^ mice were subjected to immunohistochemistry with antibodies to mouse VCAM-1; bar: 50 *μ*m. Immunoreactivity was assessed using the Image-Pro software. Results are mean ± SD of immunoreactivity signals expressed in arbitrary units; ^∗∗∗^ *p* ≤ 0.001. (b) PARP-1^−/−^ endothelial cells were transduced with an adenoviral vector encoding human PARP-1 or control virus. Protein extracts were subjected to immunoblot analysis with antibodies to PARP-1 or actin. (c) Cells from (b) were treated with 1 mg/ml LPS, 10 ng/ml TNF-*α*, or 10 ng/ml IL-1*β* for 4 h. Total RNA was then prepared, reverse-transcribed, and amplified by PCR with primer sets (Supplemental Table 1) specific to human *PARP-1*, mouse *VCAM-1*, mouse *iNOS*, or GAPDH. Amplicons were subjected to agarose electrophoresis. (d) Lung smooth muscle cells isolated from WT or PARP-1^−/−^ mice were subjected to increasing concentrations (0.01-1000 ng/ml) of LPS for 4 h. Isolated RNA was then reverse-transcribed followed by PCR with primers to mouse VCAM-1 or *β*-actin. (e) WT or PARP-1^−/−^ smooth muscle cells were treated with 100 ng/ml LPS mice and collected after 18 h. Protein extracts were subjected to immunoblot analysis with antibodies to VCAM-1 or actin. (f) PARP-1^−/−^ smooth muscle cells were transduced with the aforementioned adenoviral vectors after which cells were treated with LPS and collected 18 h later. Protein extracts were subjected to immunoblot analysis with antibodies to PARP-1, VCAM-1, or actin. (g) Protein extracts from differentiated WT, PARP-1^−/−^, or olaparib-treated WT DCs were subjected to immunoblot analysis with antibodies to VCAM-1 or actin. For (e–g), signals were quantified and are shown as values under the respective blots.

Expression of VCAM-1 on structural cells such as those of the smooth muscle also influences DC localization in inflamed tissues [40] and participates in tissue remodeling [40, 41]. We thus examined whether the effect of PARP-1 gene knockout on DC migration to the lung was associated with a reduction of VCAM-1 expression in lungs of OVA-sensitized and OVA-challenged mice.  Figure 4(a) shows that OVA-challenged mice promoted, as expected, robust expression of VCAM-1 on endothelial cells and neighboring cells, primarily smooth muscle cells. This expression was markedly reduced or completely absent in the lungs of OVA-sensitized and OVA-challenged PARP-1^−/−^ mice. We next examined whether PARP-1 is required for VCAM-1 expression in endothelial and smooth muscle cells in response to inflammatory cues. We took advantage of immortalized PARP-1^−/−^ endothelial cells and an adenoviral vector expressing human PARP-1 to conduct the experiments.  Figure 4(b) demonstrates the expression of human PARP-1 in transduced endothelial cells. The control or PARP-1-expressing cells were treated with LPS, TNF-*α*, or IL-1*β* for 4 h, and RNA isolated from the different conditions was subjected to PCR.  Figure 4(c) shows that expression of PARP-1 was required for an efficient induction of VCAM-1 in response to the tested stimuli. The results attained using TNF-*α* as a stimulus are consistent with those reported by Carrillo et al. [42]. A similar requirement for PARP-1 was observed for iNOS expression in response to IL-1*β* or LPS. TNF-*α* did not induce iNOS in these experimental conditions. When VCAM-1 expression was assessed in primary lung smooth muscle cells in response to LPS treatment, PARP-1 was also found to be required both at the mRNA (Figure 4(d)) and protein levels (Figure 4(e)). Reconstitution of PARP-1 in lung SMCs reversed VCAM-1 expression upon LPS treatment (Figure 4(f)) supporting the aforementioned results. The role of adhesion molecules on DCs is also important for their trafficking during inflammation [24]. We next examined whether PARP-1 inhibition also affected expression of VCAM-1 in fully differentiated DCs.  Figure 4(g) shows that PARP-1 inhibition by gene knockout or by olaparib severely reduced expression of VCAM-1 in DCs. These results are consistent with the effects observed in SMCs or ECs and the report by Rom et al. [43] on the role of PARP-1 in leukocyte migration through the blood-brain barrier in an *in vivo* model of localized aseptic meningitis.

It is important to emphasize the relationship between PARP-1 and VCAM-1 expressions. Although PARP-1 appears to be clearly required for the expression of the adhesion molecule in endothelial cells and lung smooth muscle cells, such requirement is absent in smooth muscle cells that are derived from the aorta. Indeed, PARP-1 gene deletion does not block expression of VCAM-1 in response to TNF-*α* treatment in vitro [44], which suggests a tissue and context-specific effect. The decrease in VCAM-1 expression upon PARP-1 inhibition may explain not only the reduction in DC migration to the lung but also that of other inflammatory cells including eosinophils, neutrophils, and macrophages. Whether PARP-1 play a critical role in eosinophil and neutrophil functions remains to be determined.

## 4. Conclusions

Overall, our results demonstrate that changes in PARP-1 protein during DC differentiation from bone marrow progenitors may constitute a dynamic process that occurs in these cells that lead to their maturation and readiness to respond to physiological and pathological cues. This conclusion is supported by the finding that PARP-1 deficiency by gene knockout or pharmacologically with olaparib does not alter DC differentiation or function. However, PARP-1 appears to play an important role in DC migration to the lung, but not to spleens and lymph nodes, upon allergen exposure. This impaired migration of DCs to the lung appears to be associated with a reduction in the expression of VCAM-1, a critical adhesion molecule for transendothelial migration of DCs. The connection between PARP-1 and VCAM-1 provides an insight on how PARP-1 inhibition reduces asthma-like traits without affecting DC function. What remains to be determined is the mechanism(s) by which PARP-1 regulates these processes. One would speculate that PARP-1 is activated by DNA damage that results from oxidative stress generated during inflammation. Such damage serves as an initiation step for the contribution of PARP-1 to inflammation. PARP-1, once activated, can posttranslationally modify transcription factors by poly(ADP-ribosyl)ation, which in turn, influences transcription of inflammatory genes. We have shown in our earlier work [22] that NF-*κ*B, which regulates expression of adhesion molecules, is modified by PARP-1 leading to its retention in the nucleus. However, this cannot be the sole mechanism by which PARP-1 regulates NF-*κ*B transcriptional activity. In response to TNF-*α*, we have reported that PARP-1 gene deletion does not affect NF-*κ*B nuclear trafficking; yet, it reduces its ability to drive expression of several key factors necessary for DC trafficking including ICAM-1, CXCR2, MCP-1, MIP-1*α*, MIP-2, and IL-8 [45]. Finally, our results suggest that PARP-1 inhibition does not cause indiscriminate immunosuppression, which represents a trait that is very important not only for inflammatory diseases but also in cancer settings.

## Data Availability

The provided data supporting the findings of this study are included within the article.

## Supplementary Materials

Supplementary Materials Table S1: primer sets for the different genes amplified by PCR. These include primer sets for human PARP-1, iNOS, and GAPDH as well as mouse VCAM-1 and *β*-actin. Figure S1: gating strategy for DC identification and phenotyping. Figure S2: gating strategy and representative histograms pertinent to the determination of T cell proliferation. Supplementary Figure S3: representative dot plots for the data displayed in Figure 2. Figure S4: percent of CD11b^+^/CD11c^+^/MHCII^interm^ cells in the different experimental groups; this is related to results displayed in Figures 2(b) and 2(c).
