# Supplementary material for: PARP-1 Is Critical for Recruitment of Dendritic Cells to the Lung in a Mouse Model of Asthma but Dispensable for Their Differentiation and Function
Source: Mediators Inflamm. 2019 Apr 24;2019:1656484. doi: 10.1155/2019/1656484 (PMC6507252; doi:10.1155/2019/1656484)

**Supplementary Table 1.** Primers sets for the different genes amplified by PCR.

| | Forward primer | Reverse primer |
|---|---|---|
| *hPARP1* | 5′-GGAAGCTGGAGGAGTGACAG-3′ | 5′-ACACCCCTTGCACGTACTTC-3′ |
| *hiNOS* | 5′-TCCTACACCACACCAAAC-3′ | 5′-CTCCAATCTCTGCCTATCC-3′ |
| *hGAPDH* | 5′-TCCCTGAGCTGAACGGGAAG-3′ | 5′-GGAGGAGTGGGTGTCGCTGT-3′ |
| | | |
| *mVCAM-1* | 5′-AGTTGGGGATTCGGTTGTTCT-3′ | 5′-CCCCTCATTCCTTACCACCC-3′ |
| *mβ-actin* | 5′-ACCGTGAAAAGATGACCCAGATC-3′ | 5′-TAGTTTCATGGATGCCACAGG-3′ |

**Supplementary Fig. S1.** Gating strategy for DC identification and phenotyping.

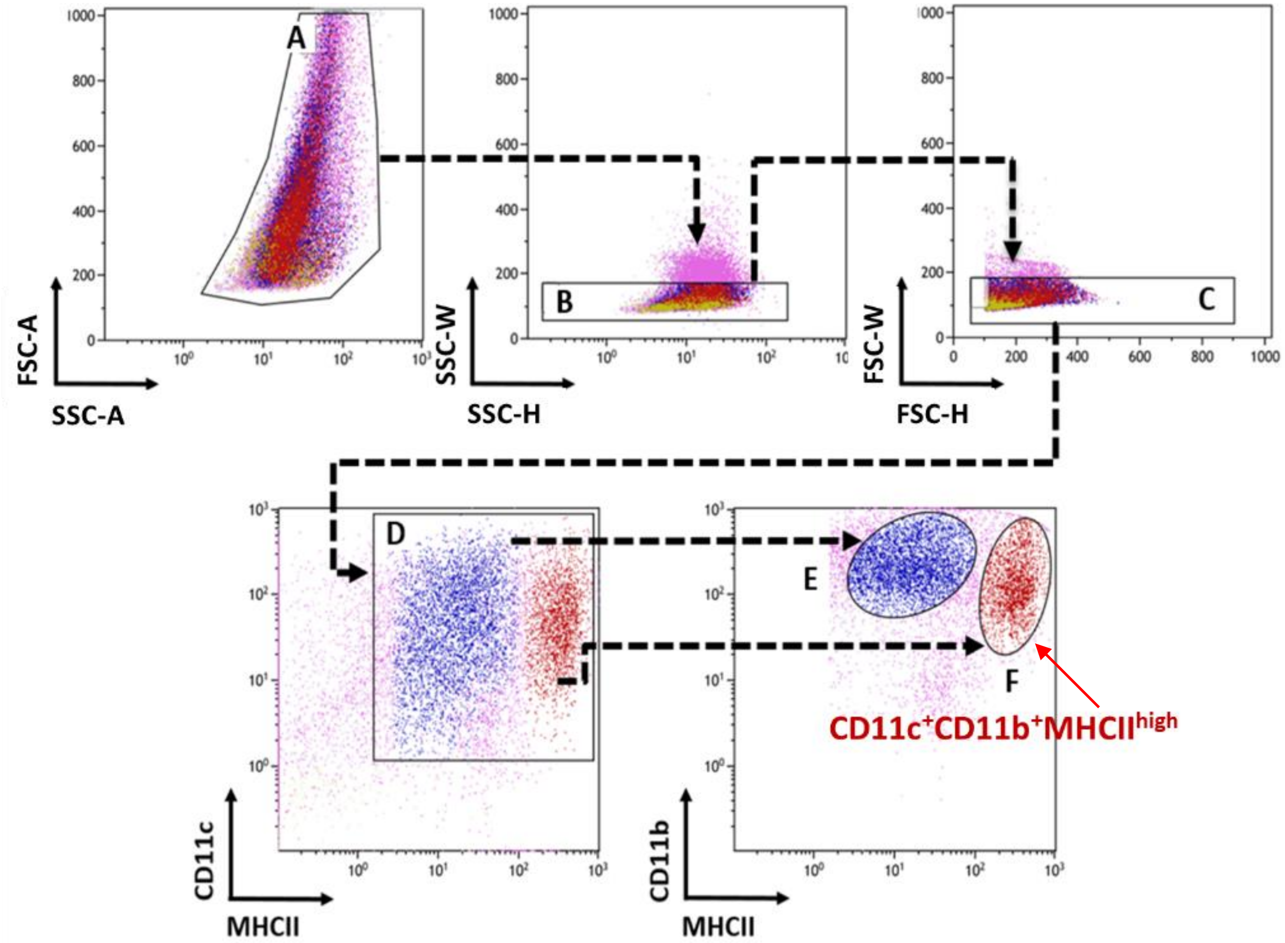

**Supplementary Fig. S2.** Gating strategy and representative histograms pertinent to the determination of T cell proliferation.

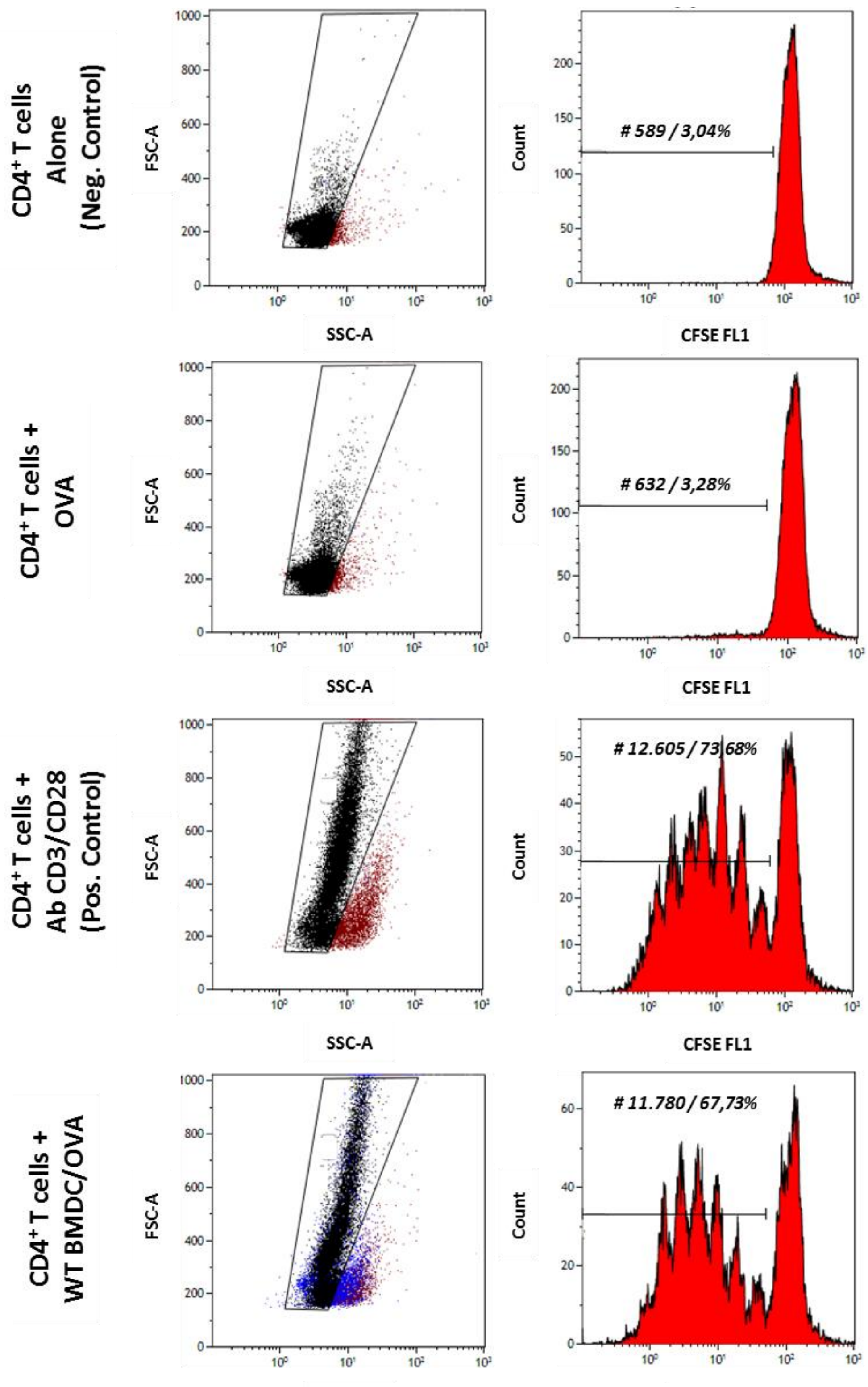

**Supplementary Fig. S3.** Representative dot plots for the data displayed in Fig. 2.

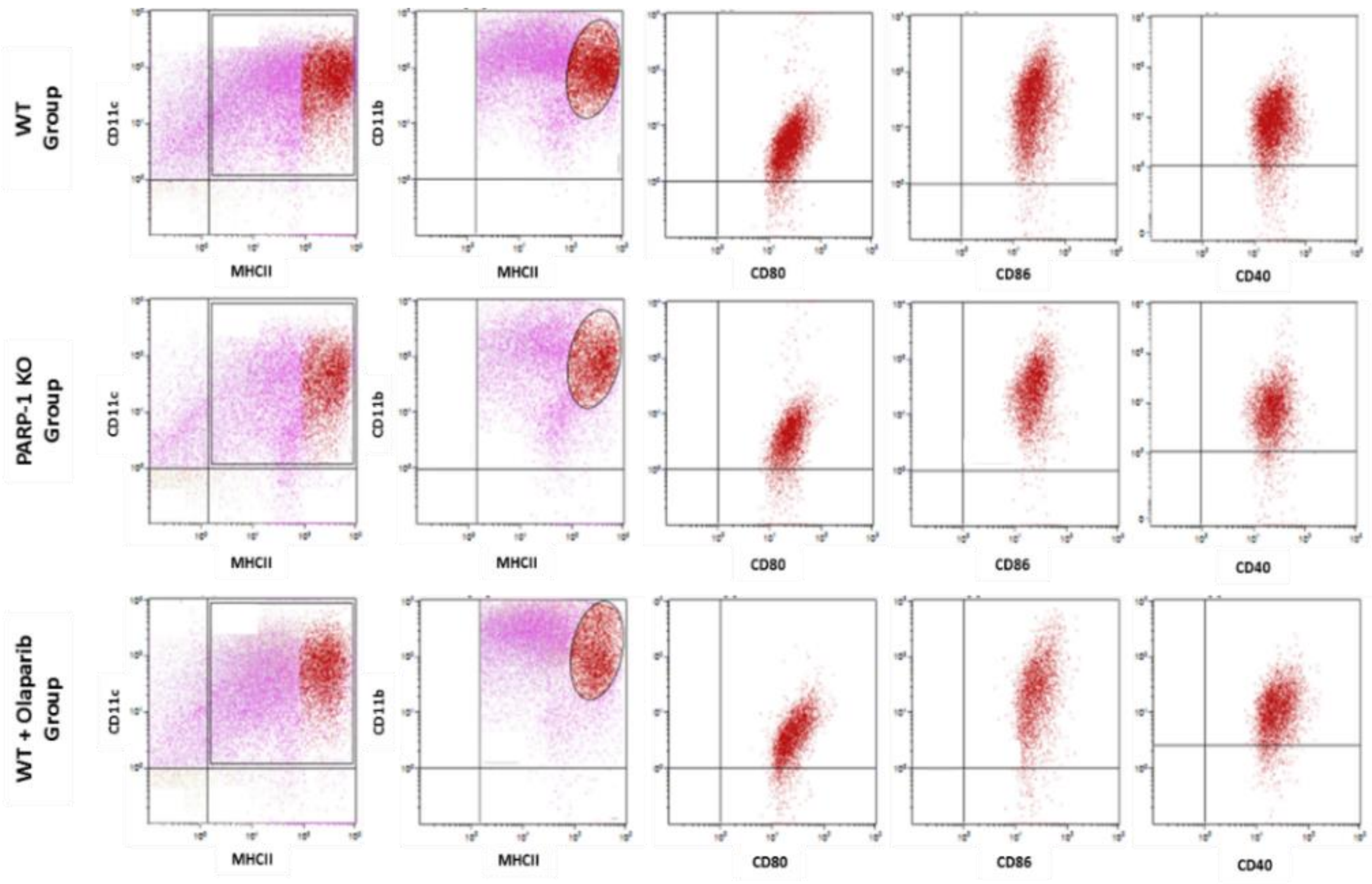


**Supplementary Fig. S4.** Percent of $CD11b^+/CD11c^+/MHCII^{interm}$ cells in the different experimental groups; this is related to results displayed in Fig.2B and 2C.

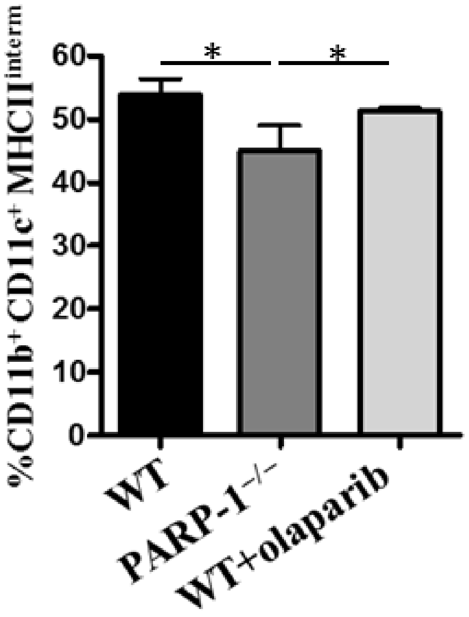

Supplement: Supplementary Materials — Table S1: primer sets for the different genes amplified by PCR. These include primer sets for human PARP-1, iNOS, and GAPDH as well as mouse VCAM-1 and β-actin. Figure S1: gating strategy for DC identification and phenotyping. Figure S2: gating strategy and representative histograms pertinent to the determination of T cell proliferation. Supplementary Figure S3: representative dot plots for the data displayed in Figure 2. Figure S4: percent of CD11b+/CD11c+/MHCIIinterm cells in the different experimental groups; this is related to results displayed in Figures 2(b) and 2(c). [file 1656484.f1.pdf]
